# Prospective acceptability, feasibility and outcomes of a primary school-led outdoor play intervention for young children

**DOI:** 10.1186/s12889-026-26846-y

**Published:** 2026-03-12

**Authors:** Amanda Louise Seims, Emily Ranken, Hanan Hauari, Laurence Ewles, Emily Midouhas, Nicola Christie, Tom Rance, Claire Cameron, Sally E Barber

**Affiliations:** 1 Bradford Teaching Hospitals Foundation Trust, Bradford Institute for Health Research, Bradford, BD9 6RJ UK; 2 Division of Health Research, Lancaster University, Lancaster, LA1 4YW UK; 3 Helen Hamlyn Centre for Pedagogy (HHCP), University College London, London, WC1E 6BT UK; 4 Thomas Coram Research Unit, University College London, London, WC1H 0NU UK; 5 Psychology and Human Development, UCL Institute of Education, University College London, London, WC1H 0AL UK; 6 Centre for Transport Studies, Department of Civil, Environmental and Geomatic Engineering, University College London, London, WC1E 6BT UK; 7 MRC Epidemiology Unit, University of Cambridge School of Medicine, Cambridge, CB2 0SL UK

**Keywords:** Child development, Urban regeneration, Children’s rights, Play sufficiency, Child-friendly cities, Whole system

## Abstract

**Background:**

Outdoor play benefits children’s mental wellbeing, social and cognitive development and physical activity levels. However, children’s outdoor play and physical activity have declined in recent years and barriers exist within their physical and social environment. Forest Schools (FS) can encourage outdoor play through familiarisation with local green spaces, but suitable spaces are limited in dense urban areas. The Play in Urban Spaces for Health (PUSH) intervention is a concept involving the use of primary school staff to support familiarisation of and regular play in local outdoor urban spaces for children aged 4–7 years. This could facilitate sustained behaviour change, whereby children use these sites with their families and have the capability and motivation to create opportunities for play within other outdoor urban spaces. The aim of this research study was to explore the prospective acceptability and feasibility of the school-led outdoor play intervention element of PUSH considering: (1) The acceptability of PUSH among primary school teaching staff and their perceived barriers and facilitators to implementation, and (2) The learning from people involved in strategic oversight and delivery of FS in urban areas, and perceived outcomes of FS.

**Methods:**

18 semi-structured interviews were conducted in Bradford, Yorkshire and Tower Hamlets, London UK using co-created topic guides: eight with teachers and headteachers at primary schools near potential urban play spaces to explore acceptability, and barriers and facilitators to potential future implementation of PUSH; and ten with staff involved in strategic oversight or delivery of FS through primary schools (either primary school staff or staff from private FS providers who are commissioned by schools) to capture learning and outcomes from taking children to local green spaces for outdoor play. Interview audio files were transcribed verbatim and coded deductively to explore acceptability using the theoretical framework of acceptability and inductively drawing on whole systems thinking to explore feasibility. This was subsequently followed by generation and revision of feasibility themes. The findings were used alongside insight from the broader PUSH research and academic literature to subsequently develop a theory of change and logic model.

**Results:**

PUSH was generally found to be acceptable. Anticipated outcomes of PUSH aligned with those perceived by participants involved in FS. Potential barriers to implementing PUSH included curriculum pressures and safety of public spaces. However, the learning from staff involved in delivering FS provided potential solutions to incorporate into the intervention design. This included linking outdoor play to the curriculum, implementing safety rules with children when at public sites, and ensuring parents are informed of where children are being taken and of the importance of outdoor play for academic learning. The findings informed the intervention theory of change and logic model for the purpose of identifying potential mechanisms of change and guiding future implementation and evaluation.

**Conclusions:**

This study offers preliminary evidence to support the delivery of the PUSH intervention within primary schools, which may support children’s habitual outdoor play. The findings emphasise the need to incorporate activities to ensure parent and staff buy-in prior to a pilot project.

## Background

The current generation of children are missing out on the potential physical, social and emotional benefits of outdoor play [1–4], and ’adventurous’ or ‘risky’ play involving situational evaluation and personal development (e.g. self-confidence and resilience) in particular [5]. Children are typically more physically active when playing outdoors [6] and engaged in unstructured play not constrained by adults [7]. However, regular outdoor play among children has dramatically declined over the past few generations [8, 9], and the average age at which children are allowed to play outside without adult supervision in Great Britain has increased by two years to age 11 compared to their parents’ generation [10]. This decline in outdoor play may partly explain why fewer than 35% of children are sufficiently active for health globally and in the UK [11, 12]. Furthermore, it may be contributing towards the reported decrease in mental well-being among children [9, 13].

Physical activity amounting to at least 180 min a day for children under 5 or an average of 60 min a day for children aged 5 or older [14] may support bone health, reduce the risk of excessive weight gain in children [15], and benefit motor skill development, cardiometabolic health [16], and emotional wellbeing [7, 17]. Across Europe, children’s levels of physical activity begin to decline around 6–7 years of age [18], highlighting the need to intervene early to reverse or delay this.

### Children’s limited access to outdoor play in urban spaces

Children have unequal access to spaces for outdoor play [19–21], with space and access typically being most limited for children living in the least affluent areas of urban settlements [19]. Furthermore, this inequality is exacerbated among children from ethnic minority groups (excluding white minority) who are more likely to live in areas deprived of greenspace than children of white ethnicity (40% vs. 14% respectively) [22]. For these children, this highlights the importance of thinking beyond green spaces as places for play. However, parents typically do not consider general urban outdoor spaces as suitable for play; many parents prefer their children to use parks and playgrounds [23], and have greater safety concerns for using urban outdoor spaces for informal play [24, 25] in comparison to spaces with traditional play equipment such as swings and slides [26].

For the global majority of children who live in urban areas [27], increasing urban densification is diminishing children’s access to places for outdoor play [28]. Significant budget cuts for local authorities in the UK have led to children’s outdoor play being deprioritised [21], and opportunities for outdoor play have diminished through the closure of a significant number of public play spaces [29–31]. These diminished and compromised opportunities for outdoor play contravene the United Nations of the Rights of the Child Article. 31 [32].

**Fig. 1:**
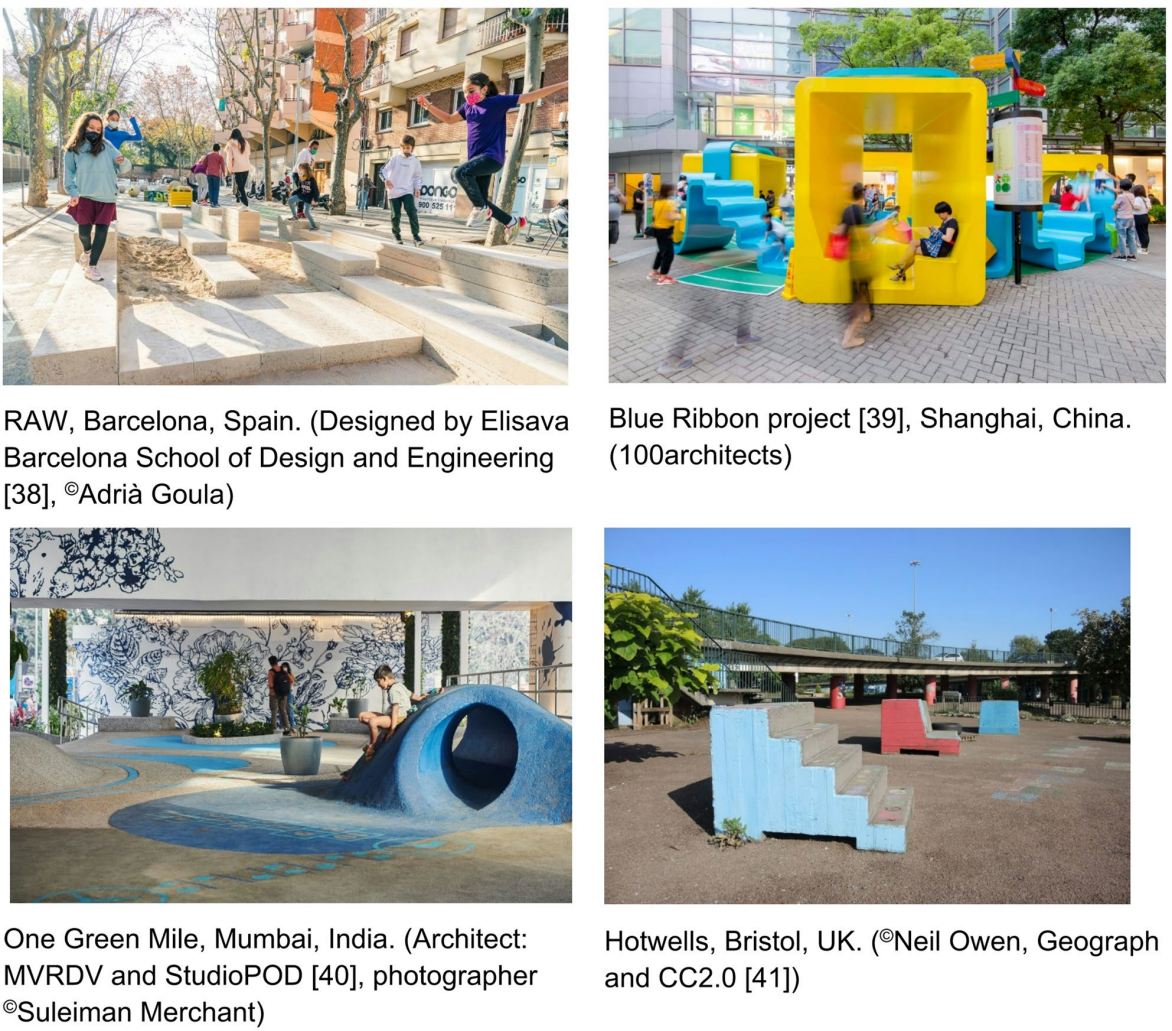
Examples of play embedded into the design of public spaces from across the globe [38–41]

The importance of outdoor play provision is increasingly recognised globally, with many cities moving towards embedding and encouraging children’s play through regenerating existing urban outdoor ‘grey’ spaces and to make them appealing for play [33–37]. In the context of this article, urban outdoor ‘grey’ spaces refer to underutilised public spaces which typically incorporate manmade materials such as concrete, tarmac or stone or small spaces. Examples of how play has been ‘designed-in’ to such spaces are shown in Fig. 1.

Such grey spaces are typically integrated within residential areas and in close proximity to places where children regularly visit, thereby being ‘familiar’ spaces and enhancing access to outdoor play by being in walkable distance from home [42]. These spaces may offer more diverse opportunities for play than green spaces and can facilitate social cohesion [42]. Despite this, there is still a need to enable and ‘normalise’ children’s play in public outdoor urban spaces through changing societal attitudes towards neighbourhood play [8, 36], parents’ perceptions of risky play [10], and antisocial behaviour and crime [25]. Taking a whole systems approach bringing various stakeholders together [43, 44] to address these physical and social environmental barriers may help unlock children’s opportunities for outdoor play in such spaces [45].

### Using schools to support children’s outdoor play

With children spending a significant amount of their waking time in school, and schools being a safe and trusted organisation for many families, the setting offers an opportunity to support outdoor play [26, 44, 46, 47]. Schools can influence physical activity behaviours beyond the school day [48] and are recognised as an important part of a systems-based approach [49]. There is also a recent recognition to include play sufficiency as a measure of school performance [29] which could enhance school buy-in to support play-based interventions.

School-led outdoor play interventions can remove some parental barriers such as lack of time or capability to support outdoor play and are reported to have parent and child support [50, 51]. Some UK schools offer Forest School (FS) which provides opportunities for children to engage in outdoor play in green spaces typically supervised by someone trained as a FS ‘leader’ and sometimes include specific learning outcomes related to the school curriculum [52]. FS is typically child-led, where children are free to pursue play which sparks their personal interest [52]. However, there are still many barriers to overcome within the school culture and staff, particularly in relation to safely supporting outdoor play which may involve risk of injury [26, 46, 53] and accommodating it within the curriculum [53, 54]. There is some evidence of acceptability and feasibility of delivering FS through primary educational settings [53–55], and of increasing children’s physical activity [56, 57]. There is emerging evidence to suggest FS influences families’ independent use of green spaces [58–60]. Given the limited access to green spaces in built-up urban areas, using other forms of urban spaces such as man-made grey or small unused green spaces should therefore be explored to facilitate child-led outdoor play within neighbourhoods.

### The Play in Urban Spaces for Health (PUSH) intervention concept

PUSH uses the same FS principle of immersive regular visits to local green spaces to influence independent outdoor play in such spaces. Unlike FS, it is anticipated that PUSH would facilitate child-led play and not include a focus on curriculum learning, allowing schools to determine how to best integrate it within the school day. PUSH is intended to promote the formation of a sustainable habit of outdoor play and incidental physical activity through familiarisation with using local urban spaces for outdoor play. PUSH was informed by the learning from previous delivery of a whole-systems, place-based children’s physical activity intervention [43, 44], the COM-B behaviour change theory [61] and the STAR ‘right to play’ framework [62]. PUSH aims to: (1) influence the regeneration of public spaces to design-in play [33–37] through understanding challenges and opportunities at the policy, strategic and environmental levels [21]; and (2) influence capabilities, opportunities and motivation in supporting children’s outdoor play among parents, children, primary school staff and the wider community. PUSH requires collaboration with key stakeholders within the local authority to identify potential urban play spaces (PUPS) where there is existing permission and funding available to design-in play, and co-design involving local children and families to develop a space. Once developed, it is anticipated that, primary schools will regularly take children (aged 4–7 years) in school time to local built-up urban spaces for child-led play to develop their capability and motivation [61] for self-initiating play, and particularly outdoor play involving physical activity and risk. The target age group was chosen to mitigate the decrease in physical activity typically observed around this age [18]. It is anticipated that, similar to FS, routine use of these types of spaces for outdoor play becomes familiar and habitual independently outside of school [58], leading to increased physical activity and wellbeing among children [56, 57], and sense of connection to the community [36]. Capturing learning from the FS delivery model and exploring school stakeholder attitudes to this approach and potential barriers and facilitators prior to a pilot may enhance the chance of successful implementation [63].

The aim of this research study was to explore the prospective acceptability, potential outcomes and feasibility of the school-led physical activity intervention element of PUSH in Bradford, Yorkshire and Tower Hamlets, London, considering: (1) the acceptability among primary school teaching staff towards PUSH, their perceived barriers and facilitators to implementation and anticipated outcomes; and (2) the learning from people involved in strategic oversight and implementation of FS in urban areas, given their similarity to PUSH. The findings informed the subsequent development of a theory of change to better understand potential mechanisms to influence whole-systems change, and a logic model to identify resources and activities needed to successfully implement PUSH, and outputs and outcomes to be captured within an evaluation. The research questions were:

1. To what extent is the PUSH school-led intervention potentially acceptable to primary school staff?
2. What factors might influence future implementation of PUSH by primary schools?
3. What positive or negative outcomes might occur through the PUSH intervention if implemented?

## Methods

### Project context and setting

The study took place from February 2023 to November 2024 across two neighbourhoods in one council ward within the city of Bradford and two areas within the London Borough of Tower Hamlets, UK. Both Tower Hamlets and the ward within Bradford are densely populated, with 16% and 11% of households being overcrowded respectively [64, 65]. In the ward within Bradford, access to green spaces and open spaces for outdoor play is a priority for regeneration [66, 67]. At the local authority level in both places, child poverty, and prevalence of overweight at primary year 6 (typically the last year of primary school in England for children aged 10–11 years old) is above the national average (Table 1).

**Table 1:** Demographic, child poverty and overweight data for Bradford, Tower Hamlets and England [68, 69]

	Bradford	Tower Hamlets	England
Total population (*N*)	546,400	310,300	56,489,800
Children aged under 16 years (% of population)	22.8	18.5	18.0
Relative child poverty after housing costs (%)	37.7	55.8	30.0
Ethnicity distribution (% non-white British)	43.3	77.1	25.6^a^
Predominant non-white British ethnic group	Pakistani	Bangladeshi	Other white
Prevalence of overweight (inc. obese) children in reception^b^ year (%)	22.3	22.0	23.0
Prevalence of overweight (inc. obese) children in primary year 6 (%)	40.8	41.8	35.2

^a^ England and Wales

^b^ first school year in England, typically when a child is aged 4–5 years old

Through partnership working with the public health and landscape design departments within the local authorities, two urban sites in built-up areas of Bradford and two in Tower Hamlets were identified as having potential or plans to regenerate for outdoor play, and these four PUPS (additional data file 1) were the focus of the research. For each space, a nearby primary school was approached via email to support the research. The Bradford primary schools incorporated a very diverse local community with ethnic groups primarily being non-white British including Indian, Polish, Russian, Nigerian, Somalian, Bangladeshi, Chinese, meaning many languages were spoken. In Tower Hamlets, one school near the PUPS served a majority Bangladeshi population.

### Study design

The study used semi-structured interviews to understand the prospective acceptability, feasibility and outcomes of the PUSH intervention using two approaches: (1) Explore acceptability of PUSH intervention among teaching staff at primary schools near the PUPS and potential barriers and facilitators to implementation; and (2) Learn from FS implementation by exploring experiences among people responsible for strategic oversight, setup and delivery of FS programmes through primary schools in Bradford and Tower Hamlets. Ethics approval was granted by University College London’s IOE Research Ethics Committee (REC1787, data protection registration number Z6364106/2023/03/71). All additional materials used to deliver the study are available through the additional data files listed in the text and accessed via OSF – see ‘availability of data and materials’ statement.

### Participants

#### Recruitment of teaching staff at primary schools near the PUPS

All primary schools located within a ~ 0.5 mile walk of each of the four PUPS were approached by email and telephone to briefly explain the purpose and nature of the research in order to recruit one school per PUPS. The first schools with staff who showed an interest in participation were sent an information sheet (additional data files 2 and 3) and the headteacher and a member of teaching staff most engaged in supporting children’s physical activity were recruited (Table 2).

#### Recruitment of staff with experience of FS oversight and delivery through urban primary schools

Local networks including private FS providers commissioned by schools, and local authority educational leads were used to identify participants who met the recruitment criteria – (1) having strategic oversight or having delivered FS programmes through primary schools in urban areas of Bradford/Tower Hamlets; (2) with delivery being part of the school curriculum inside the school day; and (3) with delivery being in public green spaces outside of the school grounds. These criteria were chosen as they closely match the delivery mode intended for PUSH, with the difference being that PUSH will be delivered in an urban public space which is not necessarily a traditional green space. Seven organisations met the criteria (including only one in Tower Hamlets) and were approached by email and telephone to briefly explain the purpose and nature of the research. Five organisations responded to take part, and an information sheet (additional files 4 and 5) was sent to primary school head teachers with experience of strategic oversight and to staff with experience of delivering FS. Through this process, ten people were recruited: nine from primary schools and one from a private FS provider in Bradford (Table 2).

All participants were given the opportunity to ask questions to the researcher via telephone or email before providing written informed consent to take part (additional data file 6).

**Table 2:** Type and number of participants recruited for interviews

Participant type	Bradford	Tower Hamlets
Head teachers with strategic oversight of FSs	4	1
FS delivery staff	4	1
PUPS teaching staff	2	2
PUPS headteachers	2	2

*PUPS * Potential urban play spaces

### Data collection - interviews

Semi-structured interviews were conducted by AS, ER, HH and LE. All interviewers were knowledgeable in acceptability and implementation frameworks and children’s health and wellbeing and had prior training and experience in interviewing.

Interviews were completed online or face to face within the organisation’s premises in a private room and typically lasted 30–50 min. Audio was recorded using a Dictaphone. Organisations participating in the research received £50 per staff member for their support with recruitment and use of time and premises to conduct the interviews.

All interviewers used the same interview topic guides that had been co-created by AS, ER, HH, SB, CC, NC and ER for the purpose of this study. Interview topic guides were created for headteachers who had strategic oversight of FS programmes (additional data file 7) and staff involved in delivery of FSs (additional data file 8) to understand the process of setup and delivery their experienced barriers and facilitators and their perceived outcomes of FS programmes. Separate interview topic guides were created for headteachers at schools near the PUPS (additional data file 9) and for the member of staff at schools near the PUPS who was most engaged in supporting children’s play and physical activity (additional data file 10) to explore acceptability, potential feasibility and their anticipated outcomes of PUSH. It was anticipated that interview participants would draw on previous experiences of school-led support of children’s outdoor play and of taking children to urban public green and grey spaces.

### Analysis of interviews

Audio recordings of interviews were imported into Microsoft Word and transcribed verbatim using the dictation mode, then checked for errors, revised, and anonymised. Transcripts were imported into NVivo (version 14.23.3, Liviero, Denver, USA).

For acceptability of PUSH, Sekhon’s Theoretical Framework of Acceptability [70] was used to guide deductive coding of data relating to perceived acceptability of PUSH (coded by AS and ER) and reported acceptability of FS (coded by AS, ER and LE). The acceptability constructs within the framework included affective attitude, burden, ethicality, intervention coherence, opportunity costs, perceived effectiveness, and self-efficacy. Drawing on whole systems thinking [43, 44], data were separately coded inductively by AS, LE and ER to identify barriers and facilitators to potential implementation of PUSH and experienced implementation of FS. All coded data were thematically analysed using a process of: (1) familiarisation with the data by AS and ER; (2) coding by AS, ER and LE as described above (3) discussion and revision of codes between AS, LE, HH and ER; (4) drawing similar and supporting data across FS and PUSH interviews into one framework by AS, ER and LE and; (5) generation of feasibility themes by AS and review and revision of themes by AS, LE, HH and ER.

Direct quotes from interviews with staff in Bradford (Bfd) and Tower Hamlets (TH) at schools near the PUPS were coded using the PUPS location number (1 or 2), and direct quotes from participants at schools delivering FSs coded using the FS location number.

### Development of the theory of change and logic model

The findings for potential acceptability, feasibility and outcomes of PUSH from this study were reviewed alongside findings from the wider PUSH project exploring acceptability and feasibility among strategic and public stakeholders (including planning and public health professionals, children, parents, and local community groups), local policy in Tower Hamlets and Bradford [21] and existing literature. These were reviewed by AS, ER, HH, CC, NC, EM and SB, and used to co-design the theory of change and logic model.

## Results

### Acceptability of PUSH

#### Affective attitude

Participants were largely positive about potentially implementing the PUSH intervention, seeing an intervention to support children’s outdoor play as ‘*worthwhile*’ and something schools ‘*needed to do*’. They implied a willingness to incorporate it as part of the school day, stating that “*it’d be absolutely something that we timetable*” (Bfd PUPS2 teacher), provided the PUPS was implemented: “*if the resource were there*,* we would use it*” (TH PUPS1 teacher).

One teacher in Bradford (Bfd PUPS1 teacher) agreed that the 4–7 years age group PUSH was targeting was suitable, as “*[young children] would love things like that…they’ve got a wild imagination*,* so they can just make things up*” (Bfd PUPS1 teacher). Staff in Tower Hamlets recognised that children across the 4–7 age range would have differing needs and interests in a play space, and that the intervention would need to be designed to be suitable for all children.

*“Just thinking of the younger children*,* things that are quite sensory… But I think as they get a bit older they do enjoy a bit more challenge… So I think it’s trying to get that balance of things that are suitable to the younger ones that are engaging in sensory [play]*,* but then things that are challenging for your older children.”* (TH teacher PUPS 2)

#### Potential effectiveness of PUSH

Staff suggested that PUSH had the potential to support children’s physical, emotional and social development, and ability to learn, and help develop their connection to place through use of PUSH sites with families. The perceived benefits of FS provided some support for these.

Bradford FS teachers perceived that children had developed “*physical skills and stamina*”, through their ability to manage the walk to and from the FS site and being “*actually able to go on long walks because of the stamina [child’s name] built up from being out in the woods*” (Bfd FS4 teacher, referring to a comment they had received from a parent). Physical improvements were particularly notable among children with previously limited physical abilities:

“*they [the pupils] may have mobility issues such as dyspraxia [developmental co-ordination disorder] or things like that. But then being outside and walking on uneven ground*,* perhaps using things like hammock swings and maintaining that proprioception*,* that spatial awareness*,* so you’ll see the development.*” (Bfd FS3 teacher)

“*[…] we also had one [pupil] that used to be carried to school every day and cry. That was last year. And by the end of Forest School*,* he was running he was loving to run into school.*” (Bfd FS4 teacher).

Schools near the PUPS felt PUSH could offer the opportunity to “*have a go at things*” and would support children to become more resilient and gain a sense of independence and “*belief in themselves”*. This was an important element of FS which was embedded in “*understanding where your limits are and that you can push out of your comfort zone and then achieve things and then push a bit further*”. Furthermore, respondents felt that children would be able to build the skills of *“planning things and breaking down problems into sets of steps and all of the communication and social stuff that can be developed through play”* (TH PUPS1 headteacher). Bradford PUPS1 teacher felt this could also enhance self-efficacy among children with additional needs:

“*a lot of the [SEN] children…their learning is through play and interaction and finding ways to sort of manage themselves so I think they would get a lot out of it.*” (Bfd PUPS1 headteacher)

Similar outcomes had been perceived by staff at a school with FS provision, who described “*that focus and that resilience and that that willingness to give it a go without panicking about not achieving a particular outcome*” (Bfd FS3 teacher), and the “*confidence to have a go with something*,* take a risk with their learning*” (Bfd FS3 co-headteacher 1) that had developed as a result of children’s participation in FS. The headteacher of a school with FS provision in Tower Hamlets also described the *“motivation and resilience”* (TH FS1 headteacher) that they had witnessed develop in children.

Staff viewed children’s participation in PUSH as a valuable opportunity for them to spend time with peers outside of the classroom, as a means of “*encouraging them to play with each other rather than be glued to a device”* (TH PUPS1 headteacher). It was suggested that this may support social cohesion through “*more interaction*,* more teamwork*,* working with each other*” (Bfd PUPS2 teacher) and “*talking to each other and learning how to be with each other*” (TH PUPS1 Headteacher) which may lead to enhanced collaborative working in the classroom:

“*…that opportunity would undoubtedly then impact on how they interact with each other in the classroom and how they’re able to apply themselves to their work or projects together.*” (TH PUPS1 Headteacher)

PUSH being part of the school curriculum was perceived as a valuable opportunity to provide children with a mental break from learning through “*getting some fresh air*,* stepping away from like the four walls of the classroom*” (Bfd PUPS1 teacher). The FS lead in TH described how children felt “relaxed” when they returned to class after having engaged in similar activities.

It was also recognised that PUSH could benefit staff-pupil relationships through “*having those discussions with the children outside of the classroom setting and that bonding time*” (Bfd PUPS2 teacher). This outcome had been achieved through the delivery of FS. One headteacher described how “*[forest school is] an opportunity outside of the classroom to have those talks*,* that isn’t as formal…it’s just like a bonding process*,* really*” (Bfd FS4 headteacher).

Staff in PUPS schools predicted that these outcomes, along with the opportunity to be physically active, would improve children’s ability to engage with academic learning, and that children would be “*much more likely when they’re back in the classroom to do what you ask them to do and to want to learn*” (TH FS1 headteacher).

Positive behavioural outcomes were reported by staff involved in FSs, particularly for those children who “*might struggle in the classroom”*, as these children “*come back having had no incidents*,* being a good role model to others*” (Bfd FS3 co-headteacher 2). Another FS headteacher described how “*for some of our more challenging children it’s calmed them down”* (Bfd FS4 headteacher). In Tower Hamlets, the FS headteacher described how the positive behavioural outcomes were worth the investment of time into the intervention:

“*When I started an intervention in a class*,* the teachers always used to say*,* ‘oh*,* but you know*,* I’m going to lose 45 minutes every week’… And then usually by the end of the intervention*,* they’d say*,* ‘do you know what? I’m going to carry on doing this because that 45 minutes that I’m investing there*,* I’m actually making it up elsewhere because I don’t spend so much time dealing with behaviour issues’.*” (TH FS1 headteacher)

There was evidence to suggest that the PUSH intervention might lead to children using the spaces outside of school time with their families. The teacher from Bradford PUPS1 provided an example where children had visited destinations with parents after going there on school trips: “*‘Oh*,* I walked past the cathedral the other day’ after they’d seen it because ‘I wanted to show my mum where we’ve been’ and things like that*,* so they want to impress you in school*”. This was supported by the FS lead teacher in Tower Hamlets, who reported that a key outcome for them was that children had taken their parents to the FS site outside of school time: “*it’s to do with them knowing that the space is there*,* that they get back and use it at other times*” (TH FS1 lead teacher).

Some Bradford staff felt that PUSH could also support children to build a connection to their neighbourhood, where currently children “*literally know their home and school and that is all*” (Bfd PUPS2 teacher) to facilitate children and families’ understanding of available opportunities (“*they don’t really know what’s available. Don’t really know what’s going on what goes on*”, Bfd PUPS1 headteacher), particularly ‘*with [Bradford City of Culture] 2025 coming out*’, the city’s forthcoming year of culture. Increased connection to and knowledge of their local area was not mentioned by participants in Tower Hamlets.

#### Anticipated burden

Despite the expressed confidence in aligning PUSH with existing school practices, there were concerns across both Bradford and Tower Hamlets that delivering PUSH in addition to the existing curriculum could be challenging for staff. Particularly in Tower Hamlets, finding the time in the timetable to take children to the urban space for play was perceived as a potential burden, meaning that the intervention would “*struggle*” in comparison to other curriculum priorities. TH PUPS1 headteacher reported that “*the timetable is very tightly packed. So we struggle*,* as it is*,* to get the actual sort of number of subjects*,* the amount of time*,* the content actually taught. It’s very*,* very difficult*”.

When initially considering implementing FSs, similar concerns had been raised by senior leadership as to how FS delivery might conflict with academic teaching. One teacher in Bfd described a comment from the headteacher: “*I like your rationale*,* but actually*,* in real world practicality terms*,* how would we make sure that it doesn’t impact on our curriculum*?”, (Bfd FS1 teacher, referring to a comment from the headteacher). However, these concerns about FS had been overcome by demonstrating opportunities for learning and linking in with the curriculum:

“*…the reason I got [the outdoor enrichment programme] passed by governors and school leaders is because I made that significant link to curriculum.*” (Bfd FS1 teacher)

“…*we do find the gaps…so there’s lots of links to PSHE [personal*,* social*,* health and economic education]*,* there’s links to science*,* there’s links to PE [physical education] sometimes. So yeah*,* we kind of make it ‘legit’ in some ways that way.*” (TH FS1 teacher)

Similarly, Tower Hamlets staff at schools near the PUPS felt more favourably towards supporting school trips which linked to the curriculum and focused on learning: “*…where children go out on trips*,* it’s part of the learning of those subjects. It has to be*,* really. There isn’t much space for it to be anything else*” (TH PUPS1 headteacher), “…*I know children learn through play*,* but learning is the bit that we have to do…*” (TH PUPS2 headteacher), “*it’s just looking at how we could incorporate it into maybe to tie in with one of the subjects rather than it being something explicit on top*” (TH PUPS2 teacher).

The staff capacity needed to implement PUSH was also a concern, with headteachers highlighting the burden of additional staff needed to safely supervise children during off-site visits, noting the need to “send at least three or four adults, so *it would have to be carefully planned and it would take a lot of person power to manage*” (Bfd PUPS2 headteacher), which would depend *“very much on the class itself and the level of needs that they have*” (TH PUPS1 headteacher). This was something highlighted by some FSs where capacity was addressed by funding external practitioners to support delivery, yet illness among key members of assisting teaching staff still had the potential to compromise delivery. Schools near the PUPS sites also noted that any potential increase in staff workload may reduce staff buy-in: “*if the staff see it as something extra that they’ve got to do or plan because their days are busy as it is*,* they’re not going to buy-in*” (Bfd PUPS2 headteacher).

#### Ethicality

Participant schools near the PUPS in both locations considered physical activity in general to be important for children, viewing it as both an area for the development of individual strengths and interests (“*we’re trying to change a little bit that life isn’t all about English and maths…not all children are academically motivated*” Bfd PUPS2 headteacher). One teacher strongly believed that “*if the children have got good gross motor skills*,* then they have good fine motor skills*” (Bfd PUPS2 teacher). In Tower Hamlets one headteacher explained they believed that *“it’s important for kids to be confident in themselves and to be able to move and express themselves”* (TH PUPS2 headteacher). Schools in both areas had an extensive variety of opportunities to support children’s outdoor play and physical activity, including active breaks in class, FS, organised games and provision of equipment to support active play time, and outsourcing some PE lessons to specialist cricket and dance providers.

Although schools were risk aware due to their responsibility for children’s safety, they supported children to take risks through climbing and exploring in the playground as this environment was considered “*safe”*. One school demonstrated an existing practice of supporting outdoor play with risk, where children were supervised from a distance and staff “*don’t stop them from exploring [playground equipment] because they’re safe and they’re built for children*” and believed that “children need to learn to manage their own risk” (Bfd PUPS1 headteacher).

However, when discussing the child-led element of the PUSH intervention, there was some hesitation for letting children “*run loose and do whatever they want”*, and a desire to ensure the time was not just spent “*messing around”* and the importance of having a “*plan*” and having staff model how to use the space:

“…*initially until we get used to the equipment ourselves*,* more of like a PE lesson where somebody models and then somebody has a go and it’s very much sort of turn taking initially.*” (Bfd PUPS2 teacher)

#### Self-efficacy

The confidence to implement PUSH (“*We will make it work. If we were to continue to be involved*”, Bfd PUPS2 headteacher) appeared to stem from how it aligned with existing school practices, partly through their experience of taking children on school trips, which was described as “*really not an issue at all*” (TH PUPS2 teacher). One Bradford headteacher described the approach they would take as part of establishing the intervention:

“*[school staff would] go down [to the PUPS] and we’d look at it and talk through some actions and talk through some things we could do*,* games that can be played and what the children are getting from it…and work together on it.*” (Bfd PUPS1 headteacher)

For Bradford headteachers, it was felt the PUSH intervention could be implemented “*as part of our physical education offer*” or alongside their existing FS programme, suggesting that “*there’s no reason if half a class are out doing that. The other half could go down there [the new play space]*” (Bfd PUPS1 headteacher). One school had a current practice of taking children to a local community centre to use their outside space for play, and considered that the PUPS would “*just become another area that we use*,* and that we get used to using*” (Bfd PUPS2 headteacher), using existing staff expertise (“*we’d probably look at [existing Forest school lead] role and how she could be used and timetabled into groups from our school to the safe space*”, Bfd PUPS2 teacher). For the Tower Hamlets school near to PUPS1, the site being “*just across the road”* deemed it acceptable, and the headteacher suggested that “*there’s no reason why we couldn’t make use of that during the children’s lunchtime*” (TH PUPS1 headteacher).

#### Intervention coherence

Staff near the PUPS demonstrated an understanding of the need for the intervention through recognising the lack of local outdoor play opportunities; Bfd PUPS2 teacher stated that “*they just don’t go out because there isn’t safe spaces for them unfortunately to play”*. The role of schools in fulfilling this need was widely acknowledged:

“*[The children] don’t have gardens*,* they don’t have outdoor space*,* so the more opportunities we can give them for safe places to play and engage with child-led play*,* I think would have a massive impact on them… when they get to school and they’ve got some open space you can see how much they love it.*” (TH PUPS1 teacher)

Schools also understood PUSH as an opportunity to increase children’s time spent being physically active in addition to current provision of school-based PE (“*They’re walking to and from the [PUPS]. They’re getting that extra time…rather than just sticking to one hour of indoor PE a week*”, Bfd PUPS1 teacher) and allowing a “*less structured*” form of physical activity than PE (Bfd PUPS1 headteacher).

Staff at schools near Bradford PUPS2 indicated an understanding of how the school-led intervention might work to change children and families’ behaviours longer-term to facilitate use of the PUPS outside of the school day, recognising the need for parent support. They felt that children would ask their parents to take them to the PUPS, and the headteacher recognised how staff could support this by asking children “*Have you been to the space this weekend? What did you do*?”.

**Fig. 2:**
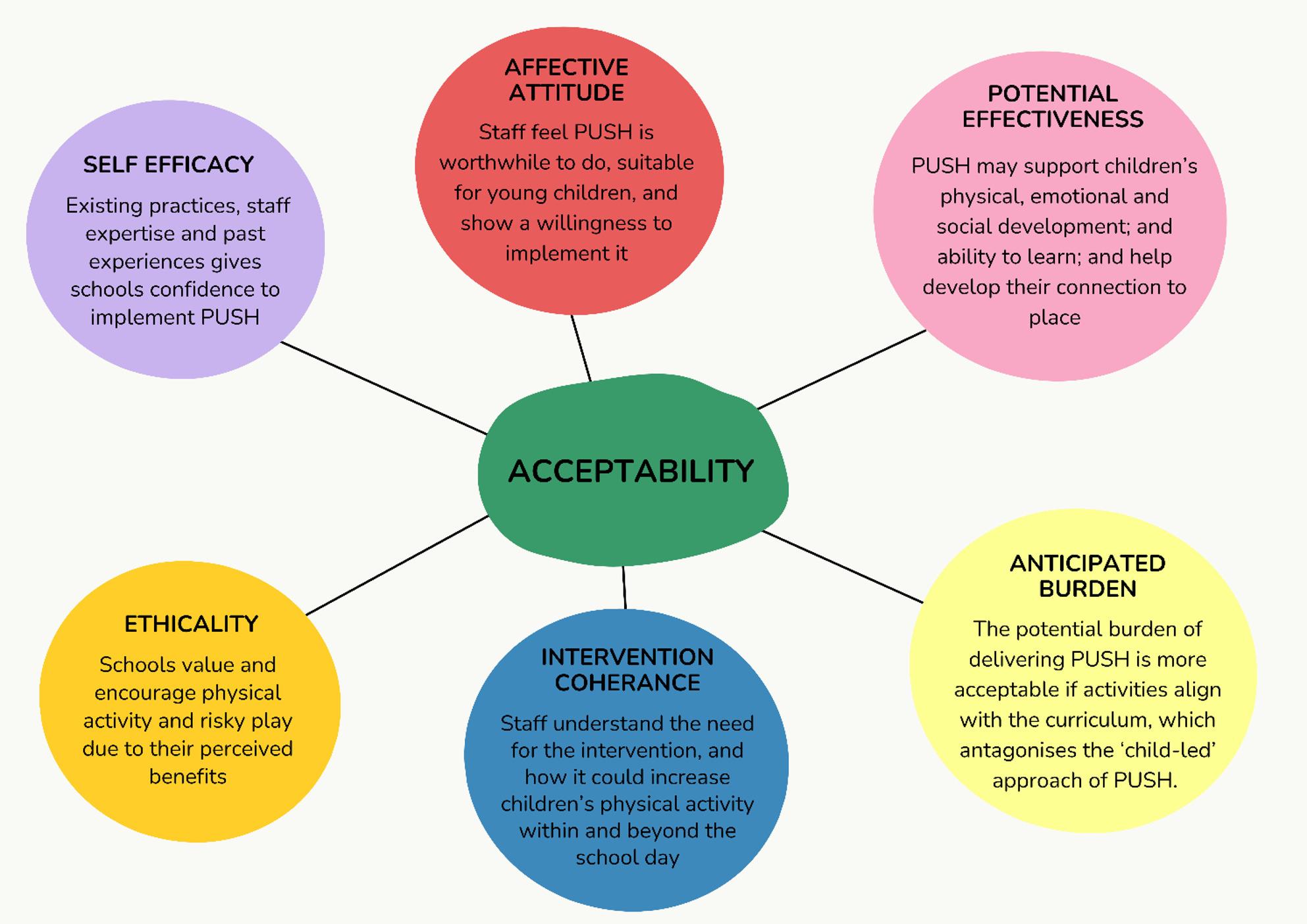
Thematic map of acceptability of PUSH

The key themes within acceptability are summarised in Fig. 2.

### Influences on the implementation of the PUSH intervention

#### Motivation and capability of staff

Schools near to the PUPS perceived that there were internal staff who would be willing to support the implementation of PUSH as they “*are really really up for things*“ (Bfd PUPS1 headteacher), are “*always wanting to learn new things and get out*“ (Bfd PUPS2 teacher), and “*like spending time outdoors*” (Bfd PUPS1 teacher) and one headteacher referred to an external sports provider who is “*always looking for opportunities to get involved in more sporting activities within the school*” (Bfd PUPS2 headteacher). A teacher in Tower Hamlets described the staff of the school as “*really dynamic*,* and we do a lot of trips*” (TH PUPS1 teacher).

It was anticipated that this willingness may be dependent on it being “*sold in the right way*” so staff understand “*why it’s so important for young children to be physically active*” (Bfd PUPS2 teacher), and the delivery being consistent through using “*the same rota of staff*,* so the staff know that they’re going to be out [of school]*” (Bfd PUPS1 teacher) and ensuring it is “*led by somebody who can have ownership…accountable to check*,* measure*,* lead*“ (Bfd PUPS2 headteacher). One headteacher in Tower Hamlets told us that it would be important to “*talk to teachers in respect of what your ideas are… so that it can really be a usable thing*” (TH PUPS1 headteacher) and to get teachers’ buy-in.

A FS headteacher reported that senior leaders had been more likely to support FS programmes if they “*100% understood the benefits to physical health*,* mental health*,* wellbeing*” (Bfd FS4 headteacher) and at another school, support from the headteacher was achieved through visiting a local school delivering FSs and “*having seen what the benefits could be for our school*” (Bfd FS3 teacher).

Both Bradford schools reported having access to staff trained in FS or play therapy (using play to help children understand and express their thoughts and feelings) or were willing to support their staff to access training opportunities using “expertise across the [school academy] trust” (Bfd PUPS1 headteacher). Staff in both Tower Hamlets and Bradford identified training needs relating to familiarisation with the intervention which could be achieved through staff visiting the spaces and “*using these types of equipment*” (Bfd PUPS2 teacher), and “*shown the possibilities*” (TH PUPS2 headteacher). This training need was also recognised by staff within FSs who referred to the importance of understanding of the “*conceptual underpinning*,* the reasons*,* the sort of pedagogy or whatever is important*” (TH FS1 teacher).

#### Existing processes and practices within schools

Existing processes within schools could support schools to take children off site for outdoor physical activity. Processes were in place to obtain parent consent for off-site visits at the point of pupil enrolment: “*we don’t have to ask for permission every time because [parents] signed for it and agreed*” (Bfd headteacher), “*anything done locally*,* we wouldn’t need to get consent for*” (TH PUPS1 teacher).

The existing practice of off-site visits meant that risk assessments have already been approved for activities of a similar nature to the PUSH intervention, with Bradford using the online ‘Evolve’ system “if we’re going on a local walk, everything tends to go on Project Evolve” (Bfd PUPS2 teacher). As part of the risk assessment for the PUPS sites, schools would still expect staff to do a pre visit to “*see where any possible dangers might be*” (Bfd PUPS1 headteacher). This was also the case in Tower Hamlets. Both Bradford schools highlighted that their learning support staff were first aid trained which was an essential requirement for enabling schools to take children off-site. Procedures to respond to emergency situations were in place across schools in Bradford and TH: “*we always take the first aid kit with us…we’d get a mobile phone for that [specific trip location]…if there was an emergency*,* they can ring the school and one of us can go and support*” (Bfd PUPS2 headteacher). “*Part of the risk assessment process would be considering all of their medical needs*,* whether they have allergies*,* whether they*,* you know*,* you need to take medication across there or not”* (TH PUPS1 headteacher).

Staff were confident of children’s ability to safely walk to PUPS through the provision of high visibility jackets and experience of walking along roads on previous trips “*knowing to stay in partners”* and were “…*generally good with walking to and from sites*” (Bfd PUPS1 teacher) and their journeys to and from school. A headteacher in Tower Hamlets described how their risk assessments might vary depending on the needs of the class:

*“You could take a class of 30 older children across there with just two adults. If you*,* if one thought that that would be safe enough*,* obviously that would depend very much on the class itself and the level of needs that they have. If you’ve got a particularly challenging class with a higher level of need*,* for example*,* then you may decide as part of that risk assessment process that you need more adults there in order to sort of safeguard the children.”* (TH PUPS1 headteacher)

Previous support from parents for off-site visits was deemed important for supporting safe staff-pupil ratios and ensuring the safe travel of children with additional needs: “…*parents have come with and supported [children with additional needs] and generally the parents have been really on board with that*” (Bfd PUPS1 headteacher).

Staff in Bradford referred to encouraging children to be “*outside all the time…regardless of the weather*” (Bfd PUPS1 headteacher) and referred to the provision of wellies and waterproofs, and asking children to “bring a sun hat, sunglasses, suncream, apply it before school. However, one school recognised that some parents struggle to provide a basic PE kit and may need schools to provide suitable clothing and equipment: “*just a pair of pumps is just an absolute nightmare…in terms of clothing*,* that could be a potential barrier if the school weren’t able to fund the children’s clothing*” (Bfd PUPS2 teacher). For FSs, the initial cost of purchasing weatherproof clothing was acknowledged, with some sourcing wellies through donations, but provision was deemed “*a massive facilitator*” where children “*were not interested in the weather*,* they’re not bothered at all*” (Bfd FS1 teacher), and clothing prevented “*scuffed skin”* from trips and falls. One school in Tower Hamlets already had *“wet weather gear for the little ones”* (TH PUPS2 class teacher) but not for the older children, who would be expected to bring their own.

#### Safety and suitability of public spaces for outdoor play

Keeping children safe outside of school was important, with one headteacher highlighting key design considerations of PUPS to address the “*need to be able to see [the children]*” when in public spaces and the importance of seating at the edges where “…*you can scan and you can see where everyone is*” (TH PUPS2 headteacher).

Despite appropriate risk assessments, it was recognised that some teaching staff may have concerns as to the fluctuating nature of the physical spaces and the people in them, referring to previous experiences of anti-social behaviour and resulting physical hazards: “*even through the day*,* [Bradford city centre] doesn’t feel like the safest place in the world…we can’t stop other people being there…I think some of our staff would be put off*” (Bfd PUPS1 teacher), “gangs are sort of hanging around [on the school premises] and then we often find like needles” (Bfd PUPS2 teacher). Similar experiences were reported by FSs who gave examples of explicit language being used, aggressive dogs off the lead and where “*somebody had gone and smashed glass purposefully and put it down the slide*” (Bfd FS2 teacher). A headteacher in Tower Hamlets expressed concerns that *“there is a different level of risk around how one uses a public space*,* because it can be accessed by anybody at any time*,* and anything could be left*” (TH PUPS2 headteacher).

Suggestions from schools near PUPS to mitigate these concerns included ensuring good links with the local community police “*making sure they are aware of what’s going on*” (Bfd PUPS2 teacher) and having an “*extra pair of eyes*” from parents or wardens (Bfd PUPS1 teacher).

Bradford FS2 and FS3 referred to the use of “*safety rules”* discussed with children at the start of each session to ensure children returned to the teacher when called (particularly if a teacher identifies a safety risk from another person): “*some kind of call and response so when you say ‘stop’ they come back to me…you have to talk about the issues with dogs and what they do if a dog comes up to them*” (Bfd FS2 teacher), “*a few kind of recall Hide and Seek games to make sure they’re maintaining the boundaries we’ve set out and they understand when to come back and things like that*” (Bfd FS3 teacher).

These risks to children’s safety were perceived as an opportunity for staff to educate children through being exposed to “*the [potential dangers] they’re going to encounter when they’re out anyway if they are out playing…there’s somebody there that can kind of support them to deal with it in the safest way*,* whether it’s*,* you know*,* antisocial dog*,* bikes*,* syringes on the floors…*” (Bfd FS3 headteacher).

Asides from safety concerns, some staff stressed the importance of ensuring the physical space accommodates children with sensory and physical needs, and some considered how facilities within or near the PUPS could support children’s ability to play outside:

“*So things like toilets*,* a place to put their shoes or their coats if they wanted to*,* you know*,* take layers off*,* for example*,* and play a bit more freely…a tap to refill their water bottles or to have access to.*” (TH PUPS2 teacher)

“*A lot of children maybe they like scoot to school*,* or they’ll bike to school. So maybe somebody that can like take a bike tour or take a scooter Yeah. So it’s got that apparatus for them to use like*,* skating area or scooting area just so they’ve got something to like*,* do.*” (Bfd PUPS1 teacher)

#### Perceived concern among parents

Schools had generally not faced many issues with parents permitting children to go on school trips, but one school gave an example where some parents doubted their child’s physical ability to walk to the local swimming baths (“*You expect them to walk?*“, “…*they can’t walk that fast*”, “*her legs hurt*”, Bfd PUPS1 headteacher referring to parent comments). Similarly, some parents at this school had been “*funny about children taking risks…*”, finding it “*quite hard to understand that you’re not following them behind all the time to catch them*” and asking “*why didn’t anyone stop them?*” when children have fallen off climbing structures in the playground (Bfd PUPS1 headteacher).

Another school referred to some parents’ beliefs that *“[their children] were going to get ill*” (Bfd PUPS2 headteacher) playing outside, and with some parents “*very*,* very protective of their children*,* don’t want their children to get wet*” (TH PUPS1 headteacher) or dirty. One school had taken the approach that “*if they’re well enough to be in school*,* they’re well enough to go*” (Bfd FS3 co-headteacher 2).

When considering the PUSH intervention, some schools were aware that some parents “*think playing is a pastime…[children are] not really learning from it*” (TH PUPS 2 headteacher) and may have concerns about children giving up time they could be spending on academic subjects “*‘why are they not doing math*,* why are they not doing English?…they’re not doing any writing’*“ highlighting the importance of educating parents on the potential benefits of outdoor play on children’s ability to engage in academic learning, as reported by FSs.

The importance of addressing parent concerns was demonstrated by some Bradford FSs, where parents typically viewed FS provision as an important enrichment experience for their children: *“The majority of the parents see the benefits that the children get out of it*” (Bfd FS3 teacher), and “*some parents have chosen us because we’ve got Forest Schools*” (Bfd FS4 headteacher). This positive support from parents was also experienced in Tower Hamlets where “*parents are really aware [Forest School is] part of the success*,* and that’s why the children do so well here”* (TH FS1 headteacher).

This success for Bradford FS4 may have been due to their efforts to address various potential concerns among parents, for example sharing photos of activities, and easing parents’ concerns by providing details of where children are going and how they are travelling. They also addressed parents’ concerns relating to exposure to cold weather through focused communication:

“…*we have got a letter or a leaflet*,* from the school nursing team*,* which basically says being cold doesn’t mean you get a cold… staff from the community…talk to parents and say*,* ‘what are you actually worried about?’.*” (Bfd FS4 headteacher)

This FS had also invited parents to experience FS which “*changed their perspective on what it is because they think they’re just going out and getting muddy and not really seeing the benefit the whole benefit of it*” (Bfd FS4 teacher) and through a focus on promoting the benefits for wellbeing: “*Lots of our parents are much more aware of mental health and its importance*” (Bfd FS4 headteacher).

**Fig. 3:**
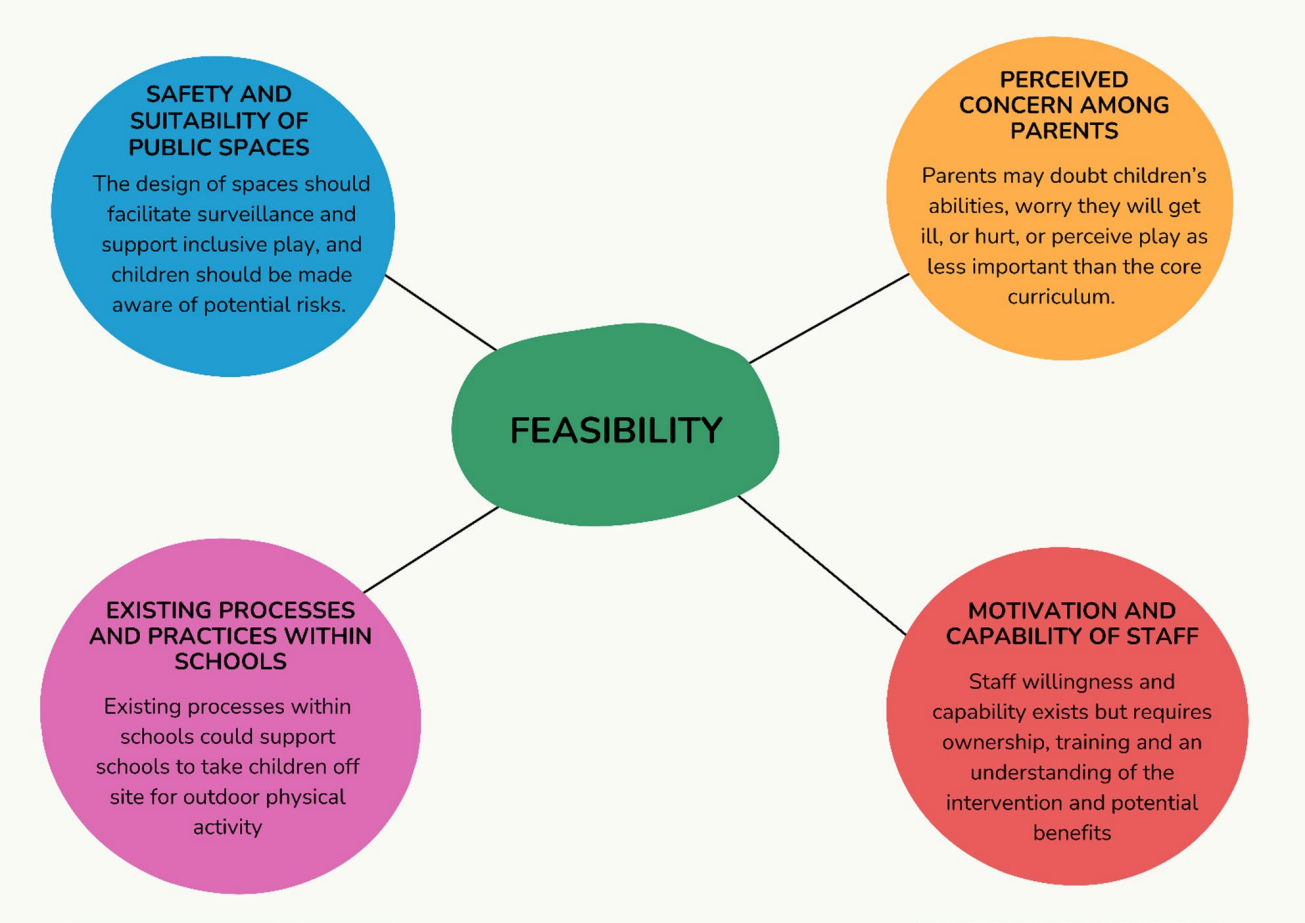
Thematic map of feasibility of PUSH

The key themes relating to potential barriers and facilitators to implementing PUSH are summarised in Fig. 3.

The co-designed theory of change and logic model developed as a result of this work and the broader research exploring acceptability and feasibility of the PUSH concept at the end-user (children and families) and strategy and policy level [21] are shown in additional data files 11 and 12.

## Discussion

This study demonstrates that primary schools could be suitable settings to facilitate outdoor play interventions such as PUSH in urban public realm spaces due to existing school cultures and practices. It also highlights key challenges to mitigate and practical additions to the intervention design which could strengthen its implementation.

### Acceptability of PUSH

The findings suggest general acceptability and coherence of the PUSH concept, with school staff anticipating many potential benefits for children, some of which had been perceived by staff delivering FS. The PUSH intervention aligned with the schools’ ethos of supporting children to have a wide range of opportunities for physically active play, meaning that PUSH was deemed acceptable by the schools involved in this study. The importance of play in school time was previously recognised by teachers during the COVID-19 restrictions [71] and previous research demonstrates acceptability of school-based unstructured active play interventions among children [72], teachers [73] and parents [74]. The perceived outcomes of PUSH have been evidenced in other school-based unstructured play interventions which reported increased fundamental movement skills [75, 76], emotional and social development [73], and educational engagement [58, 73, 77–79].

In this study, experience of FS and visiting cultural locations external to the school had led children to visit those spaces with their families outside of school time. This supports previous FS findings of connection to place and independent use of spaces [58–60], and indicates potential acceptability of PUSH among children through using informal play spaces outside of school time.

There were some concerns for the additional burden on staff and curriculum time needed to implement PUSH, which aligns with perceptions of other school-delivered outdoor play interventions [47, 52–54, 74, 76, 80]. The use of coordinators or ‘champions’ to support implementation of a similar complex intervention was highly valued by school staff [80]. Furthermore, experiences of delivering such interventions and observing benefits for children has been shown to enhance acceptability among staff and willingness to adopt this practice [76, 80], and sustainability [63]. Acceptability may be compromised in schools needing to focus on core academic subjects [80] and conditional on there being opportunities to link activities into the curriculum. However, curriculum learning through play was not the intention of PUSH and it is important that children are given the mental space away from academic pressures to pursue play [62].

### Feasibility of PUSH

PUSH was perceived as feasible to implement within existing school processes for off-site trips and staff capability, provided that early buy-in and suitable training is ensured. Presenting local health, obesity and physical activity data is perceived to enhance school buy-in [80]. Communicating the purpose, planned implementation and potential benefits of PUSH to teaching staff is important, particularly where interest in physical activity is lacking [80].

Previous research has shown that school-based delivery of outdoor play involving risk can be compromised due to some staff attitudes and concerns around health and safety [26, 55]; our research suggested staff were primarily concerned for their liability if children experienced any injuries which has been reported elsewhere in relation to play involving risk [46]. The provision of training can ensure staff understand how to safely manage this [26], and school staff have shown a willingness to undertake training to support the safe delivery of similar outdoor play interventions [55].

Despite a generally positive attitude towards supporting outdoor play involving risk among those staff interviewed, there was apprehension of the presence of the general public in urban spaces, and FS staff reported experiences of antisocial behaviour in green spaces. Using parent volunteers for greater adult presence [26], engaging the wider community to support ‘stewardship’ of the spaces and better enforcement of local antisocial behaviour regulations may mitigate this issue.

In addition to staff concerns for children’s safety, the potential of parental concerns for their child’s ability to manage risk during outdoor play is commonly reported in the literature [8, 46, 50]. The use of educational workshops with teachers and parents to ‘reframe’ risk can overcome parent and staff concern for children’s safety [26]. Parent support for outdoor play through PUSH could be facilitated through communicating the benefits in advance and having appropriate resources in place to ensure child safety [50]. As implemented by some FSs, inviting parents to volunteer at PUSH outdoor play sessions may enhance support through seeing how staff manage sessions to ensure safety and allows them to observe the benefits such as children’s enjoyment. This involvement may also provide parents with the confidence to support their child’s outdoor play at the space outside of the school day. Involving families in school-based physical activity interventions may enhance effectiveness and sustainability of children’s new skills and behaviours [81], but consideration of strategies to maintain long-term parental involvement is needed [74]. Parent concerns about children getting wet and cold are consistent with FS research [55], demonstrating the importance of schools ensuring children have access to clothing suitable for weather conditions, and of providing credible information from trusted sources such as health care professionals or community leaders to dispel myths relating to cold exposure causing viral illnesses.

### Strengths and limitations

A strength of this research is the use of the Theoretical Framework of Acceptability [70] which allowed us to understand how PUSH was acceptable among educational stakeholders. Furthermore, understanding potential barriers and facilitators to implementation may enhance the success of a future pilot through further revision of the delivery protocol. The recruitment strategy successfully enabled the completion of the targeted number of interviews in Bradford; whilst the number of interviews with FS staff in Tower Hamlets was less than intended, this was due to the lack of participants meeting the recruitment criteria. Furthermore, both schools near the Bradford PUPS had experience of delivering FS programmes and could therefore draw on their experiences of supporting children’s outdoor play within the school day at an external location. Whilst staff in those schools were confident and motivated to deliver something such as PUSH, staff in other schools without such capability, or where time for outdoor play is not deemed a priority among senior leadership, may require more buy-in, training and general support before implementation.

PUSH is a complex whole-systems intervention and the development of the theory of change through this work will facilitate its articulation to stakeholders and future research funders and includes anticipated mechanisms of behaviour change which can be evaluated during future implementation. The logic model demonstrates the need for resources and activities outside of the primary school setting to support buy-in from all stakeholders and create the right conditions overcome the challenges to implementation highlight in this study and the wider research. One example of an activity is stakeholder groups (including children, parents, school staff and the wider community) to communicate the benefits of outdoor play and importance of supporting children to play in local urban spaces. Another example is the provision of accredited training opportunities and peer support (e.g. FS leader or playworker) for school staff and parent volunteers to enhance capability to safely support child-led outdoor play. Proposed activities also include wider community education and stewardship such as the use of community groups to have oversight of spaces and the knowledge of how to report issues of antisocial behaviour. The logic model also provides a useful guide for a future process and outcome evaluation.

Limitations of this study include the potential of positive bias through recruiting the first school near a PUPS to express interest in the study. Those schools who agreed to participate typically already recognised the value of physical activity on behaviour and academic engagement. Furthermore, interviews with more strategic decision-makers at the school governance level may have revealed different attitudes towards implementation.

The research was conducted in some of the most built-up and deprived areas of Bradford and Tower Hamlets and was focused on potential implementation at specific local sites. Future studies should include other urban areas within these wards and in other cities. Evaluation of future implementation of PUSH will provide valuable insight as to the key characteristics needed to support successful delivery and outcomes.

Comparisons with previous studies was limited due to school-delivered outdoor play interventions typically being delivered within school grounds, or in green spaces which are typically considered suitable and appropriate for outdoor play. The delivery of PUSH adds complexity through the need for staff to walk children to local sites, and using sites which are not typically considered suitable or acceptable for outdoor play. However, the attitudes and concerns among staff to outdoor play within the school curriculum, and potential parent concerns were consistent with previous studies.

In this study, acceptability of PUSH among children and parents’ and their influence on its implementation were derived from participants perceptions. Acceptability and feasibility of the broader PUSH intervention among children and families attending the schools or living near the PUPS were explored through focus groups in another study (unpublished).

### Implications of findings for further development

This study provides evidence to support the acceptability and feasibility of PUSH to be implemented by primary schools, gained from insights into FS delivery and the context of schools located near the PUPS where future pilots of PUSH are anticipated. The FS insight provided useful learning to inform the delivery of PUSH and provided evidence for the potential to increase children’s physical activity and wellbeing through regular outdoor play in urban spaces. The findings demonstrate the need to adapt PUSH to include activities to ensure staff and parent buy-in prior to implementation. This could be achieved by communication strategies and parent involvement in delivery, and staff training to include experience of FSs, as used by FS participating in this research. School buy-in may also be enhanced through developing guidance on how schools might integrate PUSH within their curricula. There is also a need to explore the potential for PUSH to be delivered via community groups, initiatives and organisations which support children but are not bound to deliver an academic curriculum.

Since the research was conducted, there has been increased cross-party support to ensure children’s rights to play, including outdoor play, are enforced through play sufficiency legislation within planning, and national support for a new play strategy for England [82], demonstrating the relevance and importance of PUSH. Implementation and evaluation of evidence-based interventions such as PUSH could support the need for this strategy.

## Conclusions

Overall, our findings support the future of PUSH and highlight useful revisions to feed into its design and delivery which should enhance the success and sustainability of future implementation. PUSH aligns with a whole school approach to supporting children’s physical activity and could provide an opportunity to create behaviour change in relation to outdoor play for children living in urban areas. This could form part of the wider solution to the current problem of low levels of physical activity and wellbeing among children and aligns with the current drive to enhance children’s rights to play.

## Data Availability

The materials (listed below) used to conduct this research (participant information sheets, consent forms and interview topic guides) and the theory of change and logic model produced as an output are available online through OSF [here] (https://osf.io/7v6dq/overview?view_only=f15602325f2a446995d08043d6cf4e92) .- Additional data file 1: overview of potential urban play spaces- Additional data file 2 – participant information sheet for Bradford schools near the PUPS- Additional data file 3 - participant information sheet for Tower Hamlets schools near the PUPS- Additional data file 4 – participant information sheet for staff delivering Forest Schools in Bradford- Additional data file 5 – participant information sheet for staff delivering Forest Schools in Tower Hamlets- Additional data file 6 – consent form- Additional data file 7 – interview topic guide for headteachers of schools delivering Forest School programmes- Additional data file 8 – interview topic guide for staff delivering Forest School programmes- Additional data file 9 – interview topic guide for headteachers of schools located near the PUPS- Additional data file 10 – interview topic guide for staff supporting children’s play or physical activity at schools located near the PUPS- Additional data file 11 – theory of change- Additional data file 12 – logic modelThe datasets generated during the current study are not publicly available due to sensitivity of individual data but redacted anonymised transcripts are available from the corresponding author on reasonable request.To request access to anonymised interview transcripts, please email the Born in Bradford research team (mailto: borninbradford@bthft.nhs.uk). If your request is approved, we will ask you to sign a data sharing contract and a data sharing agreement.

## Abbreviations

Bfd: Bradford
TH: Tower Hamlets
FS: Forest School
PUSH: Play in Urban Spaces for Health
PUPS: Potential Urban Play Spaces
